# Using multi-method approaches to document and assess adaptations in a community-driven COVID-19 testing program

**DOI:** 10.1186/s43058-026-00871-9

**Published:** 2026-02-05

**Authors:** Linda Salgin, Breanna J. Reyes, Maria Balbuena Bojorquez, Angel Lomeli, Sharon Velasquez, Kelli L. Cain, Marva Seifert, Louise C. Laurent, Nicole A. Stadnick, Borsika A. Rabin

**Affiliations:** 1 San Ysidro Health, 1601 Precision Park Lane, San Ysidro, CA 92173 USA; 2 School of Public Health, San Diego State University, 5500 Campanile Drive, San Diego, CA 92182 USA; 3 Department of Obstetrics, Gynecology, and Reproductive Sciences, University of California San Diego, 9500 Gilman Dr, La Jolla, CA 92093 USA; 4 Present Address: Herbert Wertheim School of Public Health and Human Longevity Science, University of California San Diego, 9500 Gilman Dr, La Jolla, CA 92093 USA; 5 Department of Medicine, University of California San Deigo, 9500 Gilman Dr, La Jolla, CA 92093 USA; 6 Department of Psychiatry, University of California San Diego, 9500 Gilman Dr, La Jolla, CA 92093 USA; 7 Child and Adolescent Services Research Center, 6165 Greenwich Drive, Suite 300B, San Diego, CA 92122 USA; 8 Altman Clinical and Translational Research Institute, Dissemination and Implementation Science Center, University of California San Diego, 9500 Gilman Dr, La Jolla, CA 92093 USA

**Keywords:** Adaptation, COVID-19 testing, Community-based, Implementation science

## Abstract

**Background:**

Adaptations are expected when complex public health interventions are implemented in dynamically and rapidly changing real-world settings. Systematic documentation of adaptations to intervention components and strategies are critical when assessing their impact on implementation. The purpose of this paper is to describe our approach to systematically tracking, documenting, and evaluating adaptations made during the CO-CREATE-Ex project, which aimed to address COVID-19 testing disparities in the San Ysidro US/Mexico border community.

**Methods:**

The study utilized a longitudinal, prospective, multi- method approach to systematically document and assess adaptations across the pre-implementation, early and mid/late-implementation, and maintenance phases of the project. Adaptations were aggregated from a combination of sources (i.e., meeting notes, Advisory Board transcripts, and periodic reflections). Adaptations were entered weekly into an electronic database that captured information on 16 characteristics and were validated by study staff. Descriptive statistics were used to describe adaptation characteristics. Adaptation impact was evaluated using a combination of objective and subjective measures aligned with the Reach, Effectiveness, Adoption, Implementation, and Maintenance (RE-AIM) outcomes.

**Results:**

Eighty-four unique adaptations were included in this analysis. Adaptations were organized by study phase with most occurring during pre-implementation. Most adaptations (*n* = 79, 94.04%) were planned (i.e., proactive) and expected (*n* = 63, 75%), and (*n* = 21, 25.0%) adaptations were considered unexpected (e.g., reactive). Across all adaptations, 71.2% were perceived as positive (i.e., had a positive impact on RE-AIM implementation outcomes) and 19.1% were perceived to be negative (i.e., worsened implementation outcome or decreased implementation). Unexpected adaptations, though reactive in nature, generally had a positive impact on implementation outcomes. For instance, 14.3% of unexpected adaptations were perceived to increase reach and effectiveness. Within maintenance, 19% of unexpected adaptations were perceived to increase this outcome. Lastly, adaptations were generally small in scope with less than a tenth of adaptations affecting 50% or more of core elements.

**Conclusion:**

Our systematic approach to documenting and analyzing adaptations has highlighted the importance of understanding the impact of adaptations on implementation outcomes. These insights underscore the need for continued research to refine methods for adaptation documentation and impact evaluation, ensuring interventions remain effective, equitable, and responsive to real-world challenges.

**Trial registration:**

ClinicalTrials.gov, NCT05894655, Registered 8 June 2023.

Contributions to the literature

- Our study reinforces that adaptations are expected and necessary to enhance the fit of evidence-based practices to organizational contexts.
- We highlight the need for broader and more nuanced definitions of adaptation to capture both proactive and reactive changes, and to better understand their varied impacts on implementation outcomes.
- As research on evaluating adaptations remains underdeveloped, our study contributes to the literature by detailing simple yet systematic methods for documenting and assessing adaptation impact across phases of program implementation.

## Introduction

The focus on evidence-based practice in public health, social services, and medicine has elevated the importance of utilizing evidence-based practices (EBPs), with implementation science aiming to maximize the adoption, use, and maintenance of EBP’s real-world settings [1, 2]. When deploying EBPs in dynamic contexts, especially those aimed to address critical public health needs such as access to COVID-19 testing resources, adaptations are expected and necessary to enhance the fit of the EBP to the organizational context [3, 4]. Adaptions are defined as changes or modifications to an intervention or implementation strategy that aim to increase its successful implementation or effectiveness [2–4]. Adaptations can be proactive (e.g., deliberate and planned for) or reactive (e.g., in response to an unanticipated challenge) [4] and are expected when implementing programs in usual care setting [5]. Importantly, adaptations that are co-driven by researchers, community members, and organizational leaders can not only ensure that adaptations remain fidelity-consistent (i.e., adaptations that preserve core elements of an intervention) [4], but also can prevent potential inequalities in interventions by addressing the needs of underserved populations [6].

While frameworks exist to guide the general documentation and reporting of adaptations [2, 4, 7, 8], the methodologies for documenting and evaluating the impact of adaptations on implementation and effectiveness outcomes remain limited [9]. As the interest in understanding adaptations increasingly grows, analyzing their impact on critical implementation outcomes including reach, effectiveness, adoption, implementation and maintenance are needed. For example, while reactive adaptations are believed to be fidelity-inconsistent and can lead to unfavorable outcomes compared to proactive adaptations, McCarthy and colleagues found the opposite, where reactive adaptions did not lead to negative implementation outcomes [10]. Similarly, Aschbrenner and colleagues found that in their intervention, fidelity-inconsistent adaptations were positively associated with patient health outcomes, especially when those adaptations provided practical advantages [9, 10]. These findings allude to the need for systematic and detailed documentation and evaluation of adaptations as they can help identify best practices for modifying interventions and importantly increase opportunities to replicate adaptations with high impact in new settings, communities, or contexts.

### The CO-CREATE-Ex study

Community-engaged Optimization of COVID-19 Rapid Evaluation And Testing Experiences (CO-CREATE-Ex) is a two-year research study aimed to increase equitable access to COVID-19 rapid antigen tests (RATs) through the implementation of innovative, multilevel, and multicomponent implementation strategies. Details of the CO-CREATE-Ex study can be found in previous publications [11]. The study had two overarching aims; the first aim was to engage key partners in a Community and Scientific Advisory Board (CSAB) to refine and operationalize the multi-component implementation strategies and outcome metrics for COVID-19 testing. The CSAB identified i) walk-up, no-cost testing site; ii) self-service vending machines; and iii) promotora‑facilitated testing and health counseling along with preventive care gap reminders for Federally Qualified Health Center (FQHC) patients as the programs multi-component implementation strategy bundle. The second aim was to implement and evaluate the impact of the multi-component implementation strategies to optimize COVID-19 rapid testing among underserved communities using a roll-out implementation optimization (ROIO) study design [12]. The ROIO study design is a novel design that prospectively allows for iterative refinement of implementation strategies between clusters [13, 14]. Importantly, ROIO designs are particularly applicable for testing multicomponent intervention strategies which support the optimization of implementation with each successive roll-out [13, 14]. In comparison, implementation strategies in other roll-out designs, such as the stepped wedge design, remain relatively stagnant [14] potentially creating incongruence between the intervention design and organizational fit leading to fidelity-inconsistent implementation. Each of the CO-CREATE-Ex study components were rolled out sequentially across four clinic sites over the course of a year, with iterative refinements adopted prior to the subsequent roll out. These refinements identified within each roll-out were documented as adaptations to the delivery of study components. In addition, adaptations related to partner engagement via the CSAB, the overall research process, and logistical adaptations were also documented.

### Study Purpose

The primary goal of this paper is to describe our multi-method approach to systematically documenting adaptations across both intervention methods and implementation strategies as well as describing the general characteristics of adaptations. Our secondary goal was to use innovative approaches to evaluate the potential impact of adaptions on key implementation and effectiveness outcomes.

## Methods

We used a theoretically-driven, longitudinal, prospective, multi-method approach to systematically document adaptations across different phases of the CO-CREATE-Ex Study. Our documentation methodology was informed by a multi-method adaptation tracking approach that integrates elements from the original framework and coding systems for modifications and adaptations by Stirman and colleagues [15], Framework for Reporting Adaptations and Modifications to Evidence-based interventions (FRAME) [4] and the modified adaptations framework by Rabin et al. [5] This approach has been successfully applied to document adaptations across interventions, implementation strategies, and study methods in similar studies [16]. We summarized and analyzed these data to provide an overview of types of adaptations implemented at each ROIO stage (pre-implementation, implementation at each of 4 study sites, and maintenance) along with their respective impact on implementation outcomes. We assessed impact of adaptations on implementation outcomes using the reach, effectiveness, adoption, implementation, and maintenance (RE-AIM) framework [17] where we defined *Reach* as the type and number of community members engaged or reached, *Effectiveness* as any increase access to equitable testing, *Adoption* as the uptake of CO-CREATE-Ex by clinical partners, *Implementation* as acceptability of CO-CREATE-Ex for community and clinical partners, consistent and high quality delivery of testing, and costs or resources associated with testing, and *Maintenance* as ongoing delivery of consistent and high-quality testing. An additional novel measure of impact included assessing scope, based on recent work by Mark defined as the proportion of an intervention’s major subdivisions (e.g., sessions or components) that are affected by the adaptation [2, 18]. Lastly, we expand upon the traditional definitions of proactive and reactive adaptations adding additional defining characteristics leading to four operational definitions of adaptations: i) planned—where the adaptation was implemented after a discussion and consensus with at least two or more study team members using currently available data to inform decision making; ii) unplanned—where an adaptation was implemented without study team discussion and consensus, iii) expected—wherein the implementation team foresaw a necessary adaptation (e.g., translating patient facing materials from English to Arabic given the roll out of CO-CREATE-Ex among a prominent Chaldean community), or iv) unexpected—wherein an adaptation was not anticipated (e.g., temporarily shutting down operations at a site due to vending machine malfunction).

### Setting

CO-CREATE-EX was implemented at four clinic sites within San Ysidro Health (SYH), a FQHC near the US-Mexico border in San Diego County. San Ysidro Health is the county’s second largest multi-site FQHC, providing high quality and accessible services to a diverse population across with the mission to improve the health and wellbeing of the communities they serve with access for all [19]. The study is approved by the University of California, San Diego Institutional Review Board (Protocol Number 806121) and the Research Review Committee of the partnering FQHC (RHP-R-021422-86).

### Data collection and sources for adaptations

Adaptations were collected through four methods: 1) bi-weekly email reminders sent to the research and clinical teams; 2) periodic reflections; 3) meeting notes, and 4) Practical, Robust Implementation Sustainability Model (PRISM) assessments [20]. Table 1 provides an overview of the sources of the adaptations using the general guidelines for documentation of adaptations by Rabin and colleagues [5, 20]. Email reminders were developed from a previous study evaluating adaptations and refined to fit the context of CO-CREATE-Ex [16]. Staff were asked to report on any adaptations made by answering the following [4] *write a brief 1–2 sentence describing the adaption, when was this adaptation made (Day, Month, Year), When was this adaptation identified, who identified it and who made the change?* Periodic reflections [5] with research staff, clinical partners, and community partners were conducted quarterly to review the list of adaptations and add adaptations that may have been missed. The adaptations team also reviewed meeting minutes from weekly research, clinical, and community partner meetings as well as CSAB meeting recordings to retrospectively capture any adaptations. Finally, we implemented a PRISM Fit Assessment [21–23] meeting prior to the launch of CO-CREATE-Ex at each clinic site to obtain feedback from research, clinical, and community partners on programmatic implementation to identify any new adaptations. When the same barriers were identified, adaptations from earlier clinics were rolled forward to inform implementation at subsequent clinics. We also conducted a final assessment at the end of the study to determine whether barriers persisted after adaptations were adopted.

**Table 1:** Description of sources of adaptations

**Key Considerations**				
**HOW** How are data about adaptations collected?	**Source 1: Email Reminders**	**Source 2: Periodic Reflections**	**Source 3: Meeting Notes**	**Source 4: PRISM Assessment**
**WHY** Why are data about adaptations being collected?	To report any adaptations made by answering a series of questions	Reflect on current adaptations and identify any missing adaptations	Review meeting notes from all study meetings to identify adaptations not documented elsewhere	To obtain feedback on programmatic implementation and identify adaptations suggested as part of the assessment
**WHO** Who collects the data about adaptations?	Adaptation Team	Adaptation Team	Adaptation Team	Adaptation Team
**FROM WHOM** From whom are data about adaptations collected?	CO-CREATE-EX research staff	Research , clinical , and community partners	Research, clinical, and community partner meetings	Research, clinical, and community partners
**WHEN** When and how frequently are data about adaptations collected?	Bi-weekly; Email reminders start prior to the roll out of the first site during the pre-implementation phase and continue throughout the study	Quarterly; Periodic reflections begin after the roll out of the first study site and continue throughout the study	Bi-weekly; Weekly meetings notes are generated for all meetings starting at the onset of the study	Quarterly; PRISM Assessment is conducted prior to the start of each new clinic roll out
**WHAT** What type(s) of data and which characteristics of adaptations are collected?	Qualitative/Open Ended; Characteristics of adaptations	Qualitative	Qualitative	Qualitative and Quantitative
**HOW** How are data about adaptations analyzed?	Rapid qualitative analysis; Descriptive statistics	Rapid qualitative analysis; Descriptive statistics	Rapid qualitative analysis; Descriptive statistics	Rapid qualitative analysis; Descriptive statistics

An Adaptation Team consisting of two on-site study coordinators, two program managers, one student researcher, and an implementation science expert was assembled to review and discuss all adaptations on a bi-weekly basis. Once adaptations were identified, they were organized into a tracking sheet via excel. Adaptations were modified to increase clarity, combined with similar adaptations if duplicative, or removed if not considered an adaptation. Once consensus was reached by the Adaptation Team, the final version of the adaptation was entered into REDCap [24], and an adaptations matrix was developed, modeled after databases from prior studies systematically documenting adaptations [3, 10]. The adaptations matrix had nine overarching components with multiple sub-components as outlined in Table 2. Core components included: i) Partner Engagement (12 CSAB members); ii) Research Process (design [3 month rollouts, walk-up testing procedures], measures [enrollment survey], data collection [electronic surveys, PRISM Fit Assessment], analysis); iii) Logistical (IRB, translation, incentives); iv) CO-CREATE-Ex Kiosk; v) Promotores. Further details regarding these components can be found in the CO-CREATE-Ex protocol paper [11].

**Table 2:** Overview of adaptations matrix utilized to document program adaptations

Adaptations Matrix Overarching Component	Sub-Components	Definition
Who is creating this Adaptation?	Analysis/Reporter	Who is reporting adaptation in REDCap
	Source of adaptation	Who identified adaptation, may be same as Analyst/Reporter)
	Site code	- Clinic 1 - Clinic 2 - Clinic 3 - Clinic 4 - Project wide adaptation (i.e., changed made to the vending machine or role of the navigators) - CSAB - Other
	Date recorded	Date when record was made
	Date of adaptation	When adaptation was implemented
Adaptation information	Adaptation title	Title of adaptation
	Adaptation brief summary	Summary of what was adapted
	What is planned or unplanned?	Planned—change discussed with team & made decision based on data/experience Unplanned—change was made without shared discussion and agreement and possibly without looking at data
	Was it expected or unexpected?	Expected—implementation team foresaw a necessary adaptation Unexpected – implementation team did not anticipate the adaption
	Was this adaptation a result of external factors or internal issues?	External factors—related to non-research team/processes. Internal issues—related to research team/processes.
Details About Adaptation	What element was adapted?	- The setting - The format (e.g., in-person changed to telephone) - Personnel involved - The target population - How the intervention/program is presented/delivered (i.e., how core components are operationalized) - Other
	What was the type of adaptation?	- Tailoring to individuals - Adding a component (i.e., from codebook, adding materials/area of focus, adding another treatment) - Removing a component - Condensing a component (i.e., from codebook, shortening protocol from 12 to 8 sessions) - Extending a component - Substituting for a component - Changing the order of components - Repeating a component - Integrating with other programs what we are doing - Loosening the structure or protocol - Otherwise changing the intervention - Other
	Which core component is this adaptation related to?	- Partner Engagement (CSAB) - Research process (design, measures, data collection, analysis) - Logistical (IRB, translation, incentives, etc.) - CO-CREATE-Ex Kiosk - Promotores/Navigation - Other
	What was the Scope?	Percent of CO-CREATE-Ex core components affected by the adaptation
Who is involved in this adaptation?	Who was responsible for indicating this adaptation?	- Team meeting - Bi-weekly PI meeting - Monday Global ARC & UCSD meeting - On-site team meeting - UCSD Research Staff - UCSD Interns - UCSD Researchers - CSAB - Promotores - SYH Research Staff - SYH Investigator(s) - SYH Provider(s) - SYH Patients/Community Members - SYH Administration/Organization Leaders
Why was this adaptation made?	When during the CO-CREATE program was this adaptation made?	- Pre-implementation - Roll out 1 - Preparation for roll out 2 - Roll out 2 - Preparation for roll out 3 - Roll out 3 - Preparation for roll out 4 - Roll out 4 - Maintenance
	Why was this adaptation made?	Reason/what prompted need for adaptation - To increase the number and/or type of community members reached/engaged (reach) - To increase equitable testing for the right people at the right time (effectiveness) - Increase the uptake of the testing program by clinical partners (adoption) - To increase the acceptability of the testing process or program for community members (implementation) - To increase the acceptability of the testing process or program for clinical partners (implementation) - To deliver testing more consistently and with high quality (implementation) - To reduce cost and resources needed associated with testing (implementation) - To support the ongoing delivery of consistent and high-quality testing (maintenance) - Other
What was the impact of this adaptation at the end of the study?	Number and/or type of community members reached/engaged (reach)	Was there an increase, decrease, no change, unable to be determined, or not applicable
	Equitable testing for the right people at the right time (effectiveness)	Same as above
	Uptake of the testing program by clinical partners (adoption)	Same as above
	Acceptability of the testing process or program for community members (implementation)	Same as above
	Acceptability of the testing process or program for clinical partners (implementation)	Same as above
	Delivery of testing more consistently and with high quality (implementation)	Same as above
	Cost and resources needed associated with testing (implementation)	Same as above
	Ongoing delivery of consistent and high-quality testing (maintenance)	Same as above
Was this adaptation successful?	Was this adaptation successfully implemented?	Yes/No

#### Analysis

Adaptation data was analyzed using descriptive analysis. We summed the overall number of adaptations implemented throughout the study and organized and described adaptations by key adaptation characteristics (e.g., type of change, reason for change, planned, unplanned, expected, unexpected etc.). Scope of adaptations was estimated as the percentage of CO-CREATE-Ex components affected by a given adaptation. Using a study developed scale [16], the Adaptations Team evaluated the perceived impact of adaptations on key RE-AIM outcomes assessing whether the adaptation resulted in an increase, decrease, no change in a given dimension or if the given impact outcome was not able to be determined or not applicable to the adaptation. When possible, objective measures derived from available study data (e.g., participants enrolled, tests distributed, survey completion rates, costs etc.) were used to assess impact. When objective measures were not available, subjective measures (e.g., front-line staff experience, clinician reported feedback) were used. Additionally, we note that not every adaptation was intended to be evaluated for each RE-AIM outcome, and in some cases, were not intended to affect any outcomes. For example, adaptations regarding removal of testing incentives were specifically meant to target the dimension of implementation whereas adaptations to translate materials to Arabic were intended to target the dimension of implementation and reach. In other cases, for instance, adaptations made to the research process such as changing the timing of clinic roll outs were a needed adaptation to improve fit but was not intended to impact any RE-AIM outcomes.

## Results

### Adaptations overview

**Fig. 1:**
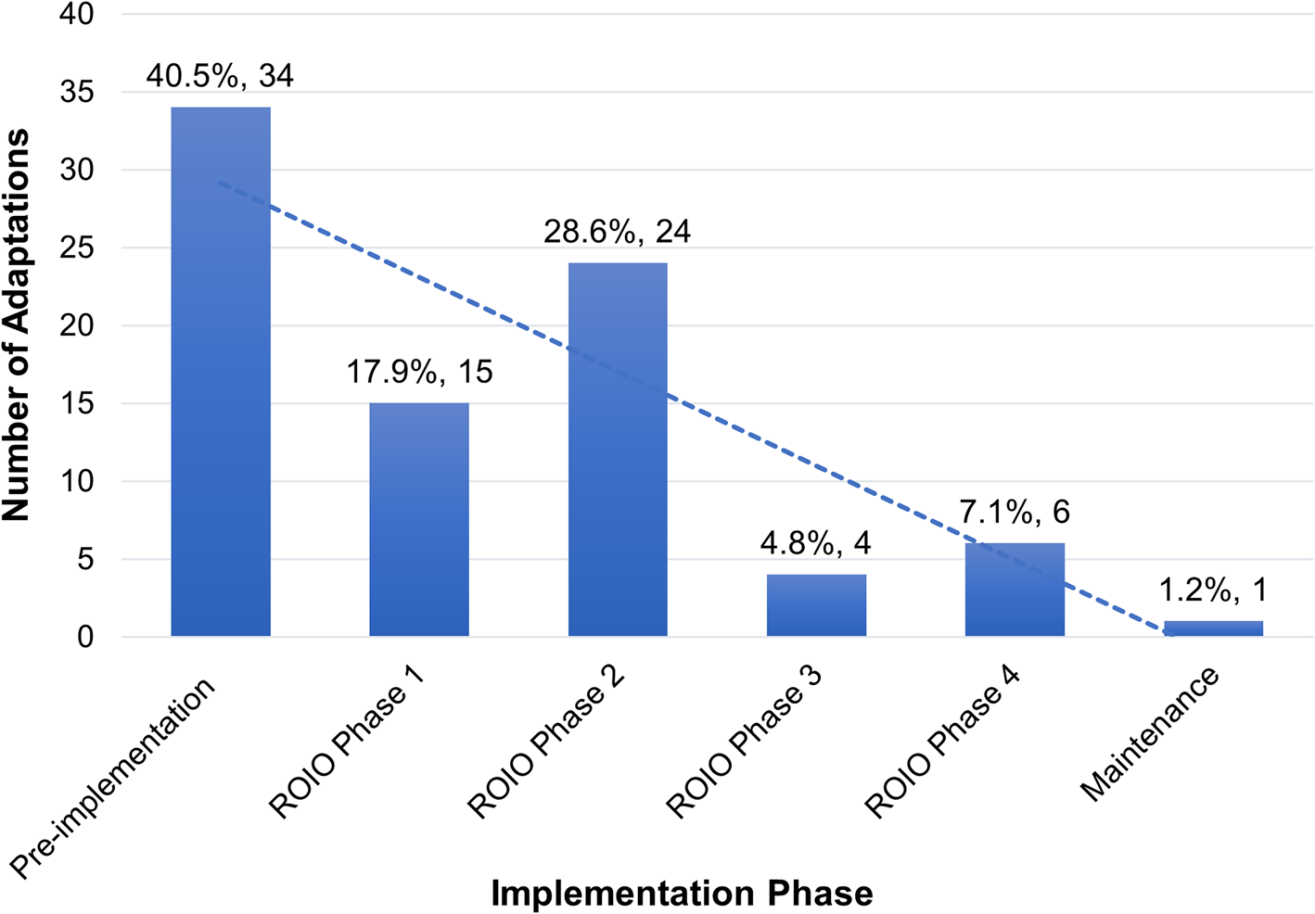
Number of unique adaptations across roll out implementation optimization study design (*n* = 84)

The Adaptations Team documented 106 adaptations. After combining duplicate entries and excluding adaptations that were not eventually implemented, 84 unique adaptations were identified and included in this analysis. Adaptations were organized in alignment to the phases of the ROIO design as seen in Fig. 1 with most adaptations occurring in the pre-implementation and early ROIO phases (i.e., roll-out 1 and 2).

The majority of adaptations (*n* = 63, 75%) were initiated by study staff who are primarily on-site, interacting daily with patients and partners. A small portion of adaptations were initiated by CSAB (*n* = 3, 3.5%), SYH administrative staff, providers, patients (*n* = 3, 3.5%) and study investigators (*n* = 9, 10.7%) (data not shown). Adaptations by ROIO phase and by adaptation constructs are represented in Table 3.

**Table 3:** Unique adaptations identified across phases of the ROIO design and adaptation constructs

Adaptation constructs	Pre-implementation		ROIO Phase 1		ROIO Phase 2		ROIO Phase 3		ROIO Phase 4		Maintenance		Total
Planned versus unplanned													
Planned	34	(43.04%)	15	(18.99%)	20	(25.32%)	4	(5.06%)	5	(6.33%)	0	(0.00%)	79
Unplanned	0	(0.00%)	0	(0.00%)	4	(80.00%)	0	(0.00%)	0	(0.00%)	1	(20.00%)	5
Expected versus unexpected													
Expected	30	(47.62%)	11	(17.46%)	17	(26.98%)	1	(1.59%)	4	(6.35%)	0	(0.00%)	63
Unexpected	4	(19.05%)	4	(19.05%)	7	(33.33%)	3	(14.29%)	2	(9.52%)	1	(4.76%)	21
Elements changed													
Setting	3	(50.00%)	1	(16.67%)	2	(33.33%)	0	(0.00%)	0	(0.00%)	0	(0.00%)	6
Format	16	(66.67%)	6	(25.00%)	1	(4.17%)	0	(0.00%)	0	(0.00%)	1	(4.17%)	24
Personnel involved	5	(62.50%)	0	(0.00%)	3	(37.50%)	0	(0.00%)	0	(0.00%)	0	(0.00%)	8
Target population	1	(50.00%)	0	(0.00%)	1	(50.00%)	0	(0.00%)	0	(0.00%)	0	(0.00%)	2
How the intervention/program is presented/delivered	9	(23.08%)	8	(20.51%)	15	(38.46%)	2	(5.13%)	5	(12.82%)	0	(0.00%)	39
Other	2	(22.22%)	1	(11.11%)	3	(33.33%)	2	(22.22%)	1	(11.11%)	0	(0.00%)	9
Type of adaptation													
Tailoring to individuals	15	(60.00%)	3	(12.00%)	6	(24.00%)	0	(0.00%)	1	(4.00%)	0	(0.00%)	25
Adding a component	13	(44.83%)	5	(17.24%)	8	(27.59%)	0	(0.00%)	3	(10.34%)	0	(0.00%)	29
Removing a component	3	(60.00%)	0	(0.00%)	1	(20.00%)	0	(0.00%)	0	(0.00%)	1	(20.00%)	5
Condensing a component	1	(33.33%)	2	(66.67%)	0	(0.00%)	0	(0.00%)	0	(0.00%)	0	(0.00%)	3
Extending a component	0	(0.00%)	1	(33.33%)	1	(33.33%)	0	(0.00%)	1	(33.33%)	0	(0.00%)	3
Substituting a component	2	(28.57%)	2	(28.57%)	1	(14.29%)	1	(14.29%)	1	(14.29%)	0	(0.00%)	7
Changing the order of components	2	(100.00%)	0	(0.00%)	0	(0.00%)	0	(0.00%)	0	(0.00%)	0	(0.00%)	2
Repeating a component	0	(0.00%)	0	(0.00%)	0	(0.00%)	0	(0.00%)	0	(0.00%)	0	(0.00%)	0
Integrating with other programs	0	(0.00%)	0	(0.00%)	0	(0.00%)	0	(0.00%)	0	(0.00%)	0	(0.00%)	0
Loosening the structure or protocol	0	(0.00%)	0	(0.00%)	0	(0.00%)	0	(0.00%)	0	(0.00%)	0	(0.00%)	0
Otherwise changing the intervention	0	(0.00%)	0	(0.00%)	0	(0.00%)	0	(0.00%)	0	(0.00%)	0	(0.00%)	0
Other	1	(14.29%)	2	(28.57%)	0	(0.00%)	3	(42.86%)	1	(14.29%)	0	(0.00%)	7
Which core component is this adaptation related to													
Partner engagement (CSAB)	2	(66.67%)	1	(33.33%)	0	(0.00%)	0	(0.00%)	0	(0.00%)	0	(0.00%)	3
Research process	22	(43.14%)	11	(21.57%)	11	(21.57%)	3	(5.88%)	3	(5.88%)	1	(1.96%)	51
Logistical	10	(31.25%)	4	(12.50%)	11	(34.38%)	1	(3.13%)	6	(18.75%)	0	(0.00%)	32
CO-CREATE-Ex kiosk	8	(42.11%)	5	(26.32%)	3	(15.79%)	1	(5.26%)	1	(5.26%)	1	(5.26%)	19
Promotores	1	(20.00%)	2	(40.00%)	2	(40.00%)	0	(0.00%)	0	(0.00%)	0	(0.00%)	5
Other	4	(66.67%)	1	(16.67%)	0	(0.00%)	1	(16.67%)	0	(0.00%)	0	(0.00%)	6
Why was the adaptation made													
To increase the number or type of patients contacted (reach)	12	(44.44%)	4	(14.81%)	9	(33.33%)	1	(3.70%)	1	(3.70%)	0	(0.00%)	27
To increase equitable testing for the right people at the right time (effectiveness)	4	(40.00%)	2	(20.00%)	2	(20.00%)	1	(10.00%)	1	(10.00%)	0	(0.00%)	10
Increase the uptake of the testing program by clinical partners (adoption)	0	(0.00%)	0	(0.00%)	2	(100.00%)	0	(0.00%)	0	(0.00%)	0	(0.00%)	2
To increase the acceptability of the testing process or program for community members (implementation)	16	(66.67%)	3	(12.50%)	3	(12.50%)	0	(0.00%)	2	(8.33%)	0	(0.00%)	24
To increase the acceptability of the testing process or program for clinical partners (implementation)	4	(36.36%)	2	(18.18%)	3	(27.27%)	0	(0.00%)	2	(18.18%)	0	(0.00%)	11
To deliver testing more consistently and with high quality (implementation)	4	(36.36%)	2	(18.18%)	4	(36.36%)	0	(0.00%)	1	(9.09%)	0	(0.00%)	11
To reduce cost and resources needed associated with testing (implementation)	4	(44.44%)	4	(44.44%)	0	(0.00%)	1	(11.11%)	0	(0.00%)	0	(0.00%)	9
To support the ongoing delivery of consistent and high-quality testing (maintenance)	5	(29.41%)	5	(29.41%)	3	(17.65%)	2	(11.76%)	2	(11.76%)	0	(0.00%)	17
Other	5	(22.73%)	3	(13.64%)	10	(45.45%)	2	(9.09%)	1	(4.55%)	1	(4.55%)	22
Was this adaptation a result of external factors or internal issues													
External	4	(28.57%)	0	(0.00%)	4	(28.57%)	4	(28.57%)	1	(7.14%)	1	(7.14%)	14
Internal	30	(42.86%)	15	(21.43%)	20	(28.57%)	0	(0.00%)	5	(7.14%)	0	(0.00%)	70

### Adaptation characteristics

#### Which core components were adapted and what was the scope of adaptations

Adaptions were made across all six core components of the CO-CREATE-Ex study. The majority of adaptations affected the core component of the research process (*n* = 51) followed by study logistics (*n* = 32), CO-CREATE-Ex vending machines (*n* = 19), other (*n* = 6), promotores (*n* = 5), and the CSAB (*n* = 3). In general, adaptations were small in scope, impacting at most half (3 out of 6) of the core components. Across all adaptations, two-thirds impacted 16.7% of core components, a quarter impacted 33.3%, and less than a tenth of adaptations impacted 50%. There was one adaptation that did not affect any core components. Table 4 presents examples of adaptations across core components including the original strategy, adaptation made, and justification for the adaptation.

**Table 4:** Examples of adaptations across CO-CREATE-Ex core components

Core Component	Original Strategy	Adaptation	Justification
*Partner Engagement (CSAB)*	12 partners would be part of the CSAB	18 partners as CSAB members	Increase diverse representation of CSAB members
*Research Process (design, measures, data collection, analysis)*	Design: ROIO design set to roll-out new clinic site every 3 months Measure: Survey to ask about general COVID19 symptoms Data Collection: PRISM Fit Assessment completed prior to each clinic roll out	Roll out between first and second clinic shortened to two 2 months and roll out of clinic 3 extended to 4 months Survey updated to include specific questions related to long covid Additional PRISM Fit assessment conducted at the end of the study	Technical challenges with Kiosk resulted in challenges launching sites in 3-month intervals Required addition by funding agency To determine if initial identified barriers persist post adaptations
*Logistical (IRB, translation, incentives)*	Translation: CO-CREATE-Ex surveys and materials offered only in English and Spanish Incentives: Participants to receive $10 for completing COVID19 RAT and $40 for completing survey	CO-CREATE-Ex surveys and materials translated into Arabic $10 testing incentives removed	Support large population of Arabic speaking patients at Kiosk location To ensure incentives are sustainable across funding period
*CO-CREATE-Ex Kiosk*	All kiosk open 24/7 once rolled out Participants will be offered both RAT and polymerase chain reaction (PCR) COVID-19 tests	Kiosk #1 temporarily shut down, limiting test availability from 8–12 pm Participants only offered RAT COVID-19 tests	Staff availability to be on-site to provide test kits To ensure sustainability of testing services
*Promotores*	Promotoras contacting participants over the phone for preventative care reminders	Promotoras additionally offering support to complete research surveys over the phone	To increase number of research surveys completed by participants

#### Planned versus unplanned

Most adaptations (*n* = 79, 94%) were planned (i.e., proactive), where the adaptation was implemented after a discussion and consensus with at least two or more study team members using currently available data to inform decision making. For instance, the team found it important to add an additional PRISM barrier assessment at the end of the study to evaluate changes between pre-implementation PRISM barrier results and post-implementation. Of those that were planned, 34 (43%) occurred during the pre-implementation phase, 44 (55.7%) during the four ROIO implementation phases, and none during maintenance. Only five adaptations were unplanned (*n* = 5, 5.9%) where an adaptation was implemented without study team discussion and consensus. For instance, survey completion was intended to be done off-site to reduce traffic at clinic entrances, but staff started providing on-site support for participants to complete their study survey in response to participant requests. Among unplanned adaptations, 4 (80%) occurred in phase two of the ROIO implementation and 1 (20%) during maintenance.

#### Expected versus unexpected

Expected adaptations accounted for 63 (75%) of the total adaptations. These adaptations included changes that the study team foresaw as a necessary adaptation for successful implementation of the study. An example of an expected adaptation was the translation of all CO-CREATE-Ex participant facing materials (e.g., flyers, consent, application) to Arabic to accommodate the predominately Arabic-speaking community served by clinic site four. Within expected adaptations, almost half (*n* = 30, 47.6%) occurred during the pre-implementation phase, 33 (52.4%) occurred during the four ROIO phases, and none during the maintenance period. In contrast, only (*n* = 21, 25.0%) of adaptations were considered unexpected (i.e., reactive). For instance, the study team did not anticipate the vending machines at clinic site two to malfunction, which then required additional on-site support (beyond the originally planned 3 months) from promotores to distribute COVID-19 testing kits. Table 5 presents the distribution of adaptations categorized by whether they were planned or unplanned, and expected or unexpected. By examining these intersections, we aimed to better understand the nature and implications of unexpected adaptations. Notably, although 21 adaptations were classified as unexpected, 85% (18/21) of those were discussed with the adaptations team (i.e., classified as planned).

**Table 5:** Distribution of adaptations characterized by planned or unplanned and expected or unexpected constructs (*n* = 84)

	Planned	Unplanned
Expected (*n* = 63)	*n* = 61, 96.8%	*n* = 2, 3.17%
Unexpected (*n* = 21)	*n* = 18, 85.7%	*n* = 3, 14.28%

#### Elements adapted & types of adaptations

Adaptations primarily were related to the element of “How the intervention/program is presented/delivered” (*n* = 39, 46.4%), followed by the “Format” (*n* = 24, 28.6%), “Personnel” (*n* = 8, 9.5%), “Setting” (*n* = 6, 7.1%), and “Target Population” (*n* = 2, 2.4%). Across all elements, 40.9% of the adaptations were during the pre-implementation phase, 57.9% across all four ROIO phases, and 1.2% during the maintenance phase. Regarding types of adaptations, of the 12 adaptation types, almost two-thirds were related to “tailoring to individuals” and “adding a component” (*n* = 25, 29.8% and *n* = 29, 34.5%, respectively).

#### Why was the adaptation made

Adaptions were made for a variety of reasons to increase reach, effectiveness, implementation, adoption, or maintenance of the Co-CREATE-Ex study. Approximately one-third (*n* = 27, 32.1%) of adaptations were made to increase reach by increasing the “number or type of patients contacted” to the CO-CREATE-Ex study followed by 24 (28.6%) adaptations which were made to increase implementation by increasing the “acceptability of the testing process or program for community members”. Adaptations to increase maintenance by supporting the “ongoing delivery of consistent and high-quality testing” accounted for 17 (20.2%) of adaptations. Regardless of reason, most adaptations occurred during the pre-implementation phase (40.5%), followed by 17.9% in ROIO phase 1, 28.6% in ROIO phase 2, 4.8% in ROIO phase 3, 7.1% in ROIO phase 4.

#### Was the adaptation due to external or internal issues

Adaptations were identified as being internal if the change derived from an issue related to the research team or process and external if resulting from an issue unrelated to the team or process. Approximately 83% of adaptations were categorized to be due to internal issues while only 16% were due to external issues. Internal issues that led to adaptations were seen more frequently in the early phases of the study with 42% in the pre-implementation phase, 21% in phase 1, and 28% in phase 2. External issues that led to adaptations were seen more consistently across phases with 4 (28.5%) adaptations in the pre-implementation phase as well as phase 2 and 3 of the study and at least 1 adaptation in phase 4 and the maintenance period of the study.

#### Impact of adaptations

Table 6 depicts the impact of adaptations across RE-AIM outcomes. Most adaptations had a positive impact on CO-CREATE-Ex implementation outcomes of Reach, Adoption, Effectiveness, Implementation, and Maintenance (i.e., an increase in the number or quality of these outcomes). Overall, across all adaptations, 56% were perceived as positive (i.e., increased implementation outcomes) and 20.4% were perceived to be negative (i.e., decreased implementation outcomes) Specifically, 17.9% of adaptations were perceived to increase Reach, 4.8% were perceived to increase Effectiveness, 2.4% to increase Adoption, 20.2% to increase Implementation, and 10.7% to increase Maintenance. Conversely, 3.6% of adaptations were perceived to reduce Reach, 3.6% were perceived to reduce Effectiveness, 12% were perceived to reduce Implementation and 1.2% were perceived to reduce Maintenance. Looking at the impact of adaptations in relation to if they were unexpected highlights that although unexpected adaptations are “reactive” in nature they generally had a positive impact on implementation outcomes. For instance, among the 21 adaptations that were unexpected, 14.3% were perceived to increase Reach while only 4.8% were perceived to reduce Reach. One adaptation was unable to be evaluated for impact while all other adaptations were not applicable to the reach dimension. Similarly, regarding Effectiveness, 14.3% of unexpected adaptations were perceived to increase Effectiveness while 4.8% were perceived to decrease Effectiveness. One adaptation was unable to be evaluated for impact while all others were not applicable to the effectiveness dimension. Only one unexpected adaptation was relevant to the adoption dimension of which its impact was unable to be determined. Within Implementation, 14.29% unexpected adaptations increased this outcome, 28.6% reduced this outcome, and 4.8% made no change in implementation. Lastly, within Maintenance (19%) of adaptations were perceived to increase Maintenance while only (4.7%) were perceived to decrease this outcome. One adaptation was unable to be determined and all others were not applicable for this dimension. Finally, while the majority of our planned (i.e., proactive) adaptations led to favorable outcomes, 2.5% were perceived to reduce Reach and 3.8% reduced Effectiveness. The largest impact was observed within Implementation, where 10.13% of planned adaptations were perceived to reduce this outcome.

**Table 6:** Impact of adaptations across RE-AIM outcomes for expected or unexpected and planned or unplanned constructs

RE-AIM Dimension	Perceived Change	All Adaptations (*n* = 84)	Expected (*n* = 63)	Unexpected (*n* = 21)	Planned (*n* = 79)	Unplanned (*n* = 5)
Reach	Increase	17.86%	19.05%	14.29%	18.99%	0.00%
	Decrease	3.57%	3.17%	4.76%	2.53%	20.00%
	No Change	1.19%	1.59%	0.00%	1.27%	0.00%
	No Applicable	64.29%	60.32%	76.19%	64.56%	60.00%
	Unable to be Determined	13.10%	15.87%	4.76%	12.66%	20.00%
Effectiveness	Increase	4.76%	1.59%	14.29%	5.06%	0.00%
	Decrease	3.57%	3.17%	4.76%	3.80%	0.00%
	No Change	2.38%	3.17%	0.00%	2.53%	0.00%
	No Applicable	83.33%	85.71%	76.19%	83.54%	80.00%
	Unable to be Determined	5.95%	6.35%	4.76%	5.06%	20.00%
Adoption	Increase	2.38%	3.17%	0.00%	2.53%	0.00%
	Decrease	0.00%	0.00%	0.00%	0.00%	0.00%
	No Change	0.00%	0.00%	0.00%	0.00%	0.00%
	No Applicable	91.67%	90.48%	95.24%	91.14%	100.00%
	Unable to be Determined	5.95%	6.35%	4.76%	6.33%	0.00%
Implementation	Increase	20.24%	23.81%	14.29%	20.25%	20.00%
	Decrease	11.90%	9.52%	28.57%	10.13%	20.00%
	No Change	7.14%	6.35%	4.76%	10.13%	0.00%
	No Applicable	38.10%	33.33%	52.38%	37.97%	40.00%
	Unable to be Determined	22.62%	26.98%	0.00%	21.52%	20.00%
Maintenance	Increase	10.71%	7.94%	19.05%	11.39%	0.00%
	Decrease	1.19%	0.00%	4.76%	0.00%	20.00%
	No Change	4.76%	4.76%	4.76%	5.06%	0.00%
	No Applicable	77.38%	79.37%	66.67%	77.22%	60.00%
	Unable to be Determined	5.95%	7.94%	4.76%	6.33%	20.00%

## Discussion

We used a theoretically driven, longitudinal, prospective, multi-method approach guided by the RE-AIM framework to systematically document and analyze adaptations across different phases of the CO-CREATE-Ex Study. This approach enabled the study team to capture adaptations in real time, which supported our understanding of how the CO-CREATE-Ex Study iteratively changed to meet the needs and priorities of our community partners and program participants alongside the evolving COVID-19 pandemic landscape. We documented a total of 84 unique adaptations with the majority being identified in the pre-implementation phase, followed by the early phases of our ROIO design, aligning to the intentions of the ROIO design which aims to integrate iterative adaptations throughout the program, with the goal of reaching optimization by the final/maintenancphase [12]. We do note that due to new funding opportunities, the CO-CREATE-Ex study began a transition process during the maintenance phase, which may have led to potential adaptations made to ensure programmatic maintenance were not captured. While adaptations were primarily identified by research study staff, our CSAB contributed to adaptation identification, which ensured that adaptations were responsive to the community. Previous studies have highlighted the important role of community advisory boards and their significant contribution to identifying programmatic priorities, participants in program study design and providing general feedback [25]. This interaction between our research study staff and community partners not only enhanced the delivery of COVID-19 tests to a highly impacted community, but also increased the successful implementation and adoption of the study overall [26, 27].

As adaptations are dynamic and nuanced, we found it important to use a combination of variables to operationalize adaptations [3, 4, 10]. Traditionally, adaptations have been classified into two categories 1) proactive—deliberately planned for, or 2) reactive – in response to an unanticipated event [4]. However, we found that these definitions can be limiting as there is a crossover between the two. For instance, an adaptation can be in response to an abrupt change that was unexpected (e.g., reactive) and can also be discussed and planned for by the study team prior to implementation (e.g., proactive). Examples of these scenarios were seen throughout our study, accounting for 21% of all adaptations, where an unexpected event occurred such as the need to shorten the time between the first and the second roll out to accommodate the anticipated surge in COVID-19 during the winter season, or changing the original location of vending machine to meet the requests of our partnering FQHC, but was able to be discussed, planned and accommodated for. As such, in this study, we created four sets of operational definitions for adaptions including adaptations that were planned for (i.e., discussed with the study team prior to implementation), unplanned (i.e., not discussed with the study team prior to implementation), expected (i.e., anticipated adaptations) and unexpected (e.g., unanticipated adaptations) allowing us to better understand the nature of the adaptations occurring within our study.

Our study addresses a significant gap in the literature on adaptations by using systematic and multi-method approaches to analyze the impact of adaptations on implementation outcomes, an area that is still growing within the field. While prior work by Stiman [4] and Miller [2] have outlined structured approaches to tracking and documenting adaptations in evidence-based interventions in the field of psychology, there is limited guidance on how to assess their impact. Similarly, while Escoffery et al., conducted a systematic review of adaptations within the field of public health describing reasons for adaptation, the adaptation process, and outcomes of adapted evidence-based interventions, they too highlight that few studies report on implementation outcomes and overall impact of adaptations [1]. These gaps highlight the need for approaches that go beyond documenting and classifying adaptations to systematically assessing their effects on key implementation outcomes, which is a key strength of this study [9].

Importantly, our operationalization of adaptations coupled with our systematic and multi-method approach to assessing impact resulted in our ability to distinguish whether or not reactive adaptations actually lead to unfavorable outcomes compared to proactive adaptations. In our study, we found that the majority of unanticipated adaptations led to positive outcomes. For instance, among the 21 adaptations that were unexpected, 14.3% were perceived to increase reach and effectiveness, while only 4.8% were perceived to reduce these outcomes. Within implementation, one unexpected adaptation (i.e., vending machine system configuration limiting COVID-19 test distribution to one per week/day versus multiple) was found to decrease the “acceptability of the testing process or program for community members” and a different unexpected adaptation (i.e., shift from using iHealth COVID-19 RAT to Genabio COVID-19 RAT) was found to decrease the “delivery of testing more consistently and with high quality”, while no adaptations were perceived to increase these outcomes. We also found that some of our planned adaptations, though proactive in nature, led to unfavorable outcomes with 2.5% of planned adaptations being perceived to reduce reach, 3.8% reduced effectiveness, and 15.9% perceived to increase “cost and resources needed associated with testing”. These results build on the findings from McCarthy and Aschbrenner whose research also found that reactive adaptations did not always lead to negative implementation outcomes and in fact can be beneficial when in response to practical needs of collaborating partners and community [9, 10].

Additional strengths of our study are the inclusion scope, a novel measure that can further elucidate the impact of an adaptation by determining the number of core components affected by an adaptation [2, 18]. In our study, regardless of adaptations being proactive or reactive, the majority were small in scope. This was expected as most adaptations were specific to a component of the study and not meant to be broad. A final strength of our study includes the use of a team-based consensus approach in determining the impact of adaptations, leveraging the collective experience of the CO-CREATE-EX study team by explicitly linking adaptations to observed implementation outcomes.

Nonetheless, our study does have limitations that should be considered. First, while we used multiple methods to identify adaptations throughout the study in real time, given the dynamism of the CO-CREATE-EX study and numerous competing demands for data collection, it is possible that not all adaptations were captured. Second, although the multi-method approaches used are comprehensive, theoretically driven, and actionable, they required significant resources from study staff and partners. While efforts were made to encourage all partners in the adaptation process such as the CSAB, SYH staff, providers, patients, as well as study investigators, adaptations were primarily initiated by staff who often were deeply involved in the implementation of CO-CREATE-Ex. These findings underscore the need for not only systematic, but also pragmatic methods for documenting adaptations to increase identification of adaptations across study partners. Thirdly, while we made meaningful strides in evaluating the impact of adaptations on a range of implementation outcomes, our assessment was limited to the end of the study period. This end-point evaluation relied on perceived consensus and retrospective review, which may introduce bias. We recognize that assessing impact iteratively throughout each stage of the ROIO design would have provided a more robust understanding of how adaptations influence outcomes over time. As the literature on evaluating impact of adaptations is still new [9, 28], our study employed innovative methods to address this gap, yet highlights the continued need for future research to develop tools, measures, and methods for quantifying the impact of adaptations on implementation outcomes across study phases. Lastly, we found that a third (*n*= 25, 29.7%) of our adaptation outcomes were unable to be determined or not applicable across any of the RE-AIM outcomes. It’s likely that our focus on assessing impact at the end of the ROIO design, rather than iteratively between each phase could have contributed to the inability to evaluate these adaptations. Additionally, in some instances, we recognized that we did not have sufficient data to determine whether or not an adaptation made an impact on implementation and effectiveness outcomes. Lastly, we would like to note that some adaptations are pragmatic in nature, and thus, are not well-positioned to be assessed for impact. These challenges further reinforce the need for refined guidelines for not only for determining which adaptations should or should not be documented, but also how different types of adaptations (e.g., adaptation of core component, intervention strategy, pragmatic adaptations) should be evaluated and emphasize the need for adaptation-outcome alignment [16].

## Conclusion

Our study integrates multiple comprehensive and theoretically driven approaches for documentation, expands on traditional definitions of adaptations, incorporates novel measures such as scope, all of which have led to a better understanding of the impact of adaptations on a wide range of implementation outcomes. Systematic documentation and evaluation of adaptations can be used to develop best practices for adapting interventions to ensure sustainable implementation in real-world contexts. Future research is needed to further develop tools, measures, and processes for analyzing adaptation impact.

## Data Availability

The datasets generated and/or analyzed during the current study are not publicly available due organizational policies, but may be available from the corresponding author on reasonable request.

## Abbreviations

CO-CREATE-Ex: Community-engaged Optimization of COVID-19 Rapid Evaluation And Testing Experiences
CSAB: Community Scientific Advisory Board
EBP: Evidence Based Practice
FRAME: Framework for Reporting Adaptations and Modifications-Enhanced
FQHC: Federally Qualified Health Center
PCR: Polymerase Chain Reaction
PRISM: Practical, Robust Implementation Sustainability Model
RAT: Rapid Antigen Tests
RE-AIM: Reach, Effectiveness, Adoption, Implementation, Maintenance
ROIO: Roll Out Implementation Optimization
SYH: San Ysidro Health
